# Mass spectrometry analysis of K63-ubiquitinated targets in response to oxidative stress

**DOI:** 10.1016/j.dib.2015.05.002

**Published:** 2015-05-21

**Authors:** Gustavo Monteiro Silva, Christine Vogel

**Affiliations:** Center for Genomics and Systems Biology, New York University, New York, NY 10003, USA

## Abstract

The data described here provide the first large-scale analysis of lysine 63 (K63)-linked polyubiquitin targets. Protein ubiquitination is a prominent post-translational modification, and a variety of ubiquitin chains exists, serving a multitude of functions [1]. The chains differ by the lysine residue by which the ubiquitin monomers are linked. We used yeast *Saccharomyces cerevisiae* subjected to oxidative stress as a model to study K63 ubiquitination. K63 ubiquitinated targets were pulled-down by the K63-TUBE system (Tandem Ubiquitin Binding Entities) and analyzed by SILAC-based mass spectrometry [2]. The data are associated to the research article ‘K63 polyubiquitination is a new modulator of the oxidative stress response’ [3]. The mass spectrometry and the analysis dataset have been deposited to the ProteomeXchange Consortium (http://proteomecentral.proteomexchange.org) via the PRIDE partner repository with the dataset identifier PXD000960.

Specifications table

**Table:** 

Subject area	Biology
More specific subject area	Yeast proteomics
Type of data	Tab-delimited and Microsoft Excel tables
How data was acquired	NanoLC 2DPlus liquid chromatography system (Eksigent), LTQ Orbitrap Velos mass spectrometer (Thermo Scientific)
Data format	Raw and MaxQuant processed files (.txt)
Experimental factors	Yeast *S. cerevisiae* cells treated with 0.6 mM H_2_O_2_
Experimental features	SILAC labeled cell culture, protein extraction, K63-ubiquitin target isolation, protein digestion, and analysis by LC–MS/MS
Data source location	New York, NY, USA
Data accessibility	Data are available via ProteomeXchange with identifier PXD000960 http://proteomecentral.proteomexchange.org/cgi/GetDataset?ID=PXD000960

Value of the data

• Largest dataset of K63-ubiquitinated proteins available to-date.
• Allows for the characterization of new targets and new potential functions for this unconventional post-translational modification.
• Quantitative analysis using SILAC-based mass spectrometry under a physiologically important condition (oxidative stress).

## 1 Data, experimental design, materials and methods

### 1.1 Experimental design

**Fig. 1:**
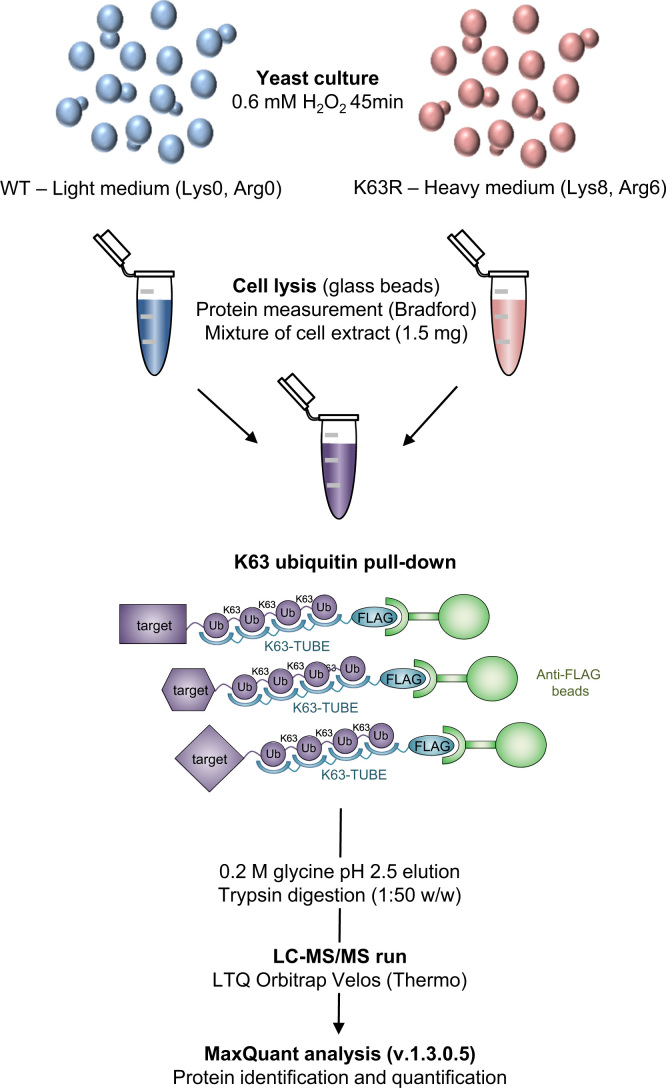
Experimental setup for the analysis of K63 ubiquitinated proteins using SILAC-based quantitative mass spectrometry.

Yeast *Saccharomyces cerevisiae* wild-type (WT) and the ubiquitin K63R mutant (K63R), which is unable to build K63 polyubiquitin chains, were subjected to H_2_O_2_ treatment to induce oxidative stress. Proteins were extracted and K63-ubiquitinated targets were pulled-down prior to analysis by high-resolution mass spectrometry. A SILAC-based mass spectrometry approach was used for quantification and differentiation between specific and unspecific interactions (Fig. 1).

### 1.2 Yeast strains and growth condition

Yeast cells were cultured in SILAC Synthetic Complete Dextrose medium depleted of arginine and lysine (0.67% yeast nitrogen base, 2% dextrose, and SC amino acid dropout without arginine and lysine from Sunrise Science). *Heavy* medium was supplemented with heavy, isotopically labeled arginine and lysine (L-Arg6 ^13^C and L-Lys8 ^13^C, ^15^N-Cambridge Isotopes), while the *Light* counterpart was supplemented with unlabeled arginine and lysine (L-Arg0 and L-Lys0). The SILAC WT and the K63R strain (GMS280 and GMS413, respectively) were derived from SUB280 and SUB413 [4] by disruption of *ARG4* and *LYS9* genes (genotype: *MATa arg4::URA3 lys9::KanMX6 lys2-801 leu2-3*,*112 ura3-52 his3-Δ200 trp1-1[am] ubi1-Δ1::TRP1 ubi2-Δ2::ura3 ubi3-Δub-2 ubi4-Δ2::LEU2 [pUB39 Ub, LYS2][pUB100, HIS3*]). Both strains express a single ubiquitin gene and have identical genotypes except for the K63R mutation (lysine 63 to arginine) in the ubiquitin gene. Cells were cultured for seven cellular divisions to ensure a high incorporation of the SILAC labels. WT and K63R cells were grown at 30 °C to log phase (OD_600_~0.5) in 50 ml of *Light* and *Heavy* medium respectively, and then treated with 0.6 mM H_2_O_2_ for 45 min prior to harvesting.

### 1.3 Protein extraction

Cells were mechanically disrupted by vortexing in the presence of 425–600 µm glass beads for three cycles of 10 min agitation at 4 °C. Lysis was performed in immunoprecipitation buffer consisting of 50 mM Tris–HCl pH 7.5, 150 mM NaCl, 5 mM EDTA, 20 mM iodoacetoamide (IAM), 1× protease inhibitor (EMD Millipore cocktail set I), and 50 nM K63-TUBE-FLAG peptide (LifeSensors). The cell extract was cleared by centrifugation (13,000×*g*) for 30 min at 4 °C. Protein concentration was determined by Bradford assay (Bio-Rad).

### 1.4 K63 ubiquitin pulldown and protein digestion

Cellular extracts were incubated for one extra hour at 4 °C to ensure thorough binding of K63-TUBE to the K63 polyubiquitin chains. Cell extracts from the WT and K63R strain (1.5 mg each) were mixed and incubated for 1 h under rotation with 50 µl Protein G-Dynabeads (Invitrogen) loaded with M2 anti-FLAG antibody (Sigma - 7 μg antibody/mg beads). Next, the beads were washed four times, and the K63 targets were eluted with 0.2 M glycine pH 2.5 for 1 h at 4 °C. Eluate was mixed at 1:1 ratio with trifluoroethanol (TFE) and reduced with 15 mM DTT for 45 min at 55 °C. Prior to cysteine alkylation with 55 mM IAM for 30 min in dark, the pH was adjusted with one volume of 1 M Tris–HCl pH 8.0 to prevent unspecific carbamidomethylation. TFE concentration was diluted to 5% with 50 mM Tris–HCl pH 8.0. Trypsin was added at 1:50 ratio (w/w) and samples were incubated overnight at 37 °C under agitation. The reaction was stopped by adding 1% formic acid, dried down to 10–20 μl by speedvac centrifugation, and cleaned up using C18 Hypersep Spin Tip (Thermo Scientific) following the manufacturer׳s instructions.

### 1.5 Mass spectrometry analysis

Peptides were separated on a 15 cm Agilent ZORBAX 300 StableBond C18 column (75 μm ID, 3.5 μm particle, 300 Å pore size) by reverse-phase chromatography with a gradient of 5 to 40% acetonitrile over 160 min on an Eksigent NanoLC 2DPlus liquid chromatography system. LC-eluted peptides were injected in-line onto an LTQ Orbitrap Velos mass spectrometer (Thermo Scientific). Data-dependent analysis was performed using the top 20 most intense ions from each MS full scan. Dynamic exclusion was set to 90 seconds if mass to charge ratio (*m/z*) acquisition was repeated within a 45 seconds interval. MS1 data was collected in the FTMS mass analyzer at 60,000 resolution, in profile mode, and automatic gain control at 1E6. MS2 data was collected in the Ion Trap mass analyzer, in centroid mode, automatic gain control at 3E4, injection time of 100 ms, isolation window of 1 *m/z,* and normalized collision energy at 35%. Samples were injected three times to obtain technical replicates. The immunoprecipitation experiment was conducted twice to obtain two biological replicates (IPK63_1 and IPK63_2). To determine significance, data were compared to that from untreated cells (IPK63_Untreated). The deposited data (PXD000960) also contain two biological replicates (WCE1 and WCE2) from a SILAC-based mass spectrometry analysis of the *W*hole *C*ell *E*xtract (cell lysate) prior to the K63 ubiquitin pulldown. The analyses compared the protein expression levels of the WT to the K63R strain, and each biological replicate was also injected three times as technical replicates to improve quantitation and coverage.

### 1.6 Data processing

The RAW data files were combined and processed using MaxQuant (version 1.3.0.5) matching against the yeast *S. cerevisiae* database (Uniprot, 2012 release). SILAC analyses were performed selecting Arg6 and Lys8 as heavy labels. Two missed cleavages were allowed, choosing trypsin as the proteolytic enzyme. Cysteine carbamidomethylation was selected as fixed, and diglycyl lysine, methionine oxidation, and N-terminal acetylation were selected as variable post-translational modification. Mass tolerance was set to 20 ppm for the FT mass analyzer and 0.5 Da for the Ion Trap mass analyzer. The false discovery rate (FDR) for protein, peptide, and site modifications was set to 1% based on the reverse yeast FASTA file. Minimum peptide length was seven, and every protein group was required to have at least one unique or razor peptide. Minimum ratio count for SILAC pairs was set to two. A complete set of parameters is provided in the MaxQuant results file deposited at ProteomeXchange. The results files (.zip) include two sets of MaxQuant tab delimited output tables (.txt) for the K63 ubiquitin immunoprecipitation (IPK63) and for the whole cell extract (WCE). The ‘protein_groups’, ‘peptides’, ‘evidence’, ‘parameters’ and ‘summary’ output files are presented here as Supplementary Tables 1–5, respectively, in Microsoft Excel spreadsheet format. Post-processing analyses eliminated contaminants and reversed sequences, and used only proteins present in both biological replicates, having at least two different peptides identified. Significance cut-off (1.57 fold change) was established based on the distribution of ratios (Light/Heavy) of the treated versus the untreated cells at 5% FDR.
